# Risk Factor Analysis Including Inflammatory Markers for ICU Admission and Survival After Pneumonectomy

**DOI:** 10.3390/medicina60111768

**Published:** 2024-10-29

**Authors:** Mediha Turktan, Ersel Gulec, Alper Avcı, Zehra Hatıpoglu, Ilker Unal

**Affiliations:** 1 Department of Anesthesiology and Reanimation, Faculty of Medicine, Cukurova University, 01330 Adana, Turkey; gulecersel@yahoo.com (E.G.); hatipogluzehra@gmail.com (Z.H.); 2 Department of Thoracic Surgery, Faculty of Medicine, Cukurova University, 01330 Adana, Turkey; dravcialper@gmail.com; 3 Department of Biostatistics, Faculty of Medicine, Cukurova University, 01330 Adana, Turkey; ilkerun@cu.edu.tr

**Keywords:** inflammatory markers, intensive care, pneumonectomy, risk factor, survival

## Abstract

*Background and Objectives:* To assess the impact of preoperative inflammatory parameters on the necessity for intensive care unit (ICU) admission and survival after pneumonectomy. *Materials and Methods:* We enrolled 207 adult patients who underwent pneumonectomy between December 2016 and January 2022. We collected data from patients’ electronic medical records. *Results:* The preoperative albumin level was statistically lower, need for blood transfusion was higher, and length of hospital stay was longer in ICU-admitted patients (*p* = 0.017, *p* = 0.020, and *p* = 0.026, respectively). In multivariate analysis, intra-pericardial pneumonectomy and postoperative complications were predictive factors for ICU admission (OR = 3.46; 95%CI: 1.45–8.23; *p* = 0.005 and OR = 5.10; 95%CI: 2.21–11.79; *p* < 0.001, respectively). Sleeve or pericardial pneumonectomy (*p* = 0.010), intraoperative vascular injury (*p* = 0.003), the need for mechanical ventilation (*p* < 0.001), acute renal failure (*p* = 0.018), sepsis (*p* = 0.008), respiratory failure (*p* < 0.001), pneumonia (*p* = 0.025), the need for blood transfusion (*p* = 0.047), elevated blood urea nitrogen (BUN) (*p* = 0.046), and elevated creatinine levels (*p* = 0.004) were more common in patients who died within 28 days. Patients who died within 90 days exhibited higher preoperative neutrophil-to-lymphocyte ratio (NLR) values (*p* = 0.019) and serum creatinine levels (*p* = 0.008), had a greater prevalence of sleeve or intra-pericardial pneumonectomy (*p* = 0.002), the need for mechanical ventilation (*p* < 0.001), intraoperative vascular injury (*p* = 0.049), sepsis (*p* < 0.001), respiratory failure (*p* = 0.019), and contralateral pneumonia (*p* = 0.008) than those who did not. *Conclusions:* Intra-pericardial pneumonectomy and postoperative complications are independent predictors of ICU admission after pneumonectomy. Tracheal sleeve and intra-pericardial procedures, intraoperative and postoperative complications, the need for blood transfusion, preoperative NLR ratio, BUN and creatinine levels may also be potential risk factors for mortality.

## 1. Introduction

Pneumonectomy, a major surgical procedure typically performed for lung cancer and involving the complete removal of a lung, continues to present challenges despite advancements in surgical and anesthetic practices. It is associated with high rates of postoperative pulmonary complications and mortality, with mortality rates ranging from 5% to 21% [1,2,3,4,5]. Studies have revealed that several factors can impact mortality and the risk of respiratory failure after pneumonectomy [2,6].

The role of inflammation in cancer is complex and multifaceted. While it was shown to inhibit tumor development by stimulating immune processes, it was also demonstrated to promote tumor growth and metastasis [7]. Parameters related to inflammation were employed to gauge the systemic inflammatory response and immune system activity. In recent years, there has been growing interest among researchers in assessing the systemic inflammatory response as a predictor of postoperative complications and mortality. The prognostic significance of novel inflammatory parameters such as lymphocyte-to-monocyte ratio (LMR), neutrophil-to-lymphocyte ratio (NLR) and platelet-to-lymphocyte ratio (PLR) was investigated in lung cancer patients and PLR was found to be an independent prognostic factor for survival in this population after lung cancer surgery [7]. However, their specific influence on the need for admission to the intensive care unit (ICU) and survival following high-risk surgeries, such as pneumonectomy, remains uncertain.

In this study, we set out to explore the potential role of serum albumin level, neutrophil-to-albumin ratio (NAR), white blood cell count (WBC), LMR, NLR, and PLR in predicting the requirement for ICU admission and survival (28 and 90 days) in patients undergoing pneumonectomy, in addition to other risk factors. The primary outcome focused on the necessity for admission to the ICU, while the secondary outcome was the 28- and 90-day survival rate after pneumonectomy.

## 2. Materials and Methods

After obtaining ethical approval from the Cukurova University Faculty of Medicine Scientific Ethics Committee (approval code: 127/29; date: 4 November 2022), this retrospective, observational and cross-sectional study utilized the hospital’s medical information system and anesthesia follow-up records. This study followed the guidelines outlined in the Declaration of Helsinki. Since it was retrospective and individual patients were not identified, informed consent was not required.

### 2.1. Study Groups

The study included 207 adult patients who underwent elective pneumonectomy for lung cancer between 31 December 2016 and 1 January 2022 at a tertiary hospital in Turkey with the same thoracic surgeon. Exclusion criteria encompassed patients below the age of 18, those with clinical indications of infection, other inflammatory conditions, or hematological disorders, planned ICU admission before surgery, as well as those with inaccessible hospital records.

### 2.2. Study Design

All patients were documented using standardized case report forms, which compiled information on demographics, smoking history, comorbidities, and laboratory values assessed one day before surgery. Laboratory values included monocyte, lymphocyte, neutrophil, and white blood cell counts, albumin, hemoglobin, hematocrit, blood urea nitrogen, and creatinine levels. The LMR, NLR, PLR, and NAR were calculated based on the aforementioned results. Surgical details, the admission to ICU within 28 days after surgery, the need for mechanical ventilation via an endotracheal tube (either continued postoperative use, or re-intubation) within 28 days after surgery, the total blood transfusion requirement (during the intraoperative and postoperative period), the length of hospital stay, and 28- and 90-day survival data were also recorded.

The criteria for admission to the ICU were grouped into five categories: close follow-up, respiratory failure, acute renal instability, postoperative cardiopulmonary arrest, and hemodynamic instability. Close follow-up was defined as admission to the ICU for monitoring purposes only, without the implementation of additional interventions (vasoactive support, ventilation support including high flow or noninvasive mechanical ventilation, etc.) in accordance with the request of the anesthesiologist or surgeon. The term “respiratory failure” was defined as the presence of any of the following criteria: shortness of breath, peripheral oxygen saturation < 90%, tachypnea (>20 breaths/min), bradypnea (<12 breaths/min), hypoxia (partial pressure of oxygen < 60 mmHg), hypercapnia (partial pressure of carbon dioxide > 50 mmHg), and confusion. Hemodynamic instability was defined as a state of hypotension requiring the use of vasopressors. Acute renal failure was defined as a serum creatinine concentration exceeding 4 mg/dL or a three-fold increase in preoperative values.

### 2.3. Statistical Analysis

Statistical analysis was conducted using the IBM SPSS Statistics software package program (IBM Corp., Released 2011. IBM SPSS Statistics for Windows, Version 20.0. Armonk, NY, USA: IBM Corp). Categorical variables were presented as counts or percentages, while numeric variables were described using mean and standard deviation (or, if applicable, median and interquartile range: IQRs). Categorical measurements were compared using either the Pearson Chi-square test or the Fisher Exact test, depending on appropriateness. The normality of data distribution was assessed using the Shapiro–Wilk test. For normally distributed data, the independent samples *t*-test was employed, and the Mann–Whitney U test was used for variables exhibiting non-normal distribution. The Wilcoxon Signed Rank test was applied to compare two dependent numerical measures that did not follow a normal distribution. Logistic regression analysis was performed to determine significant predictors of ICU admission. In univariate analysis, variables significant at the *p* < 0.25 level were entered in logistic regression analysis. The significance level for all statistical tests was set at 0.05.

## 3. Results

**Figure 1:**
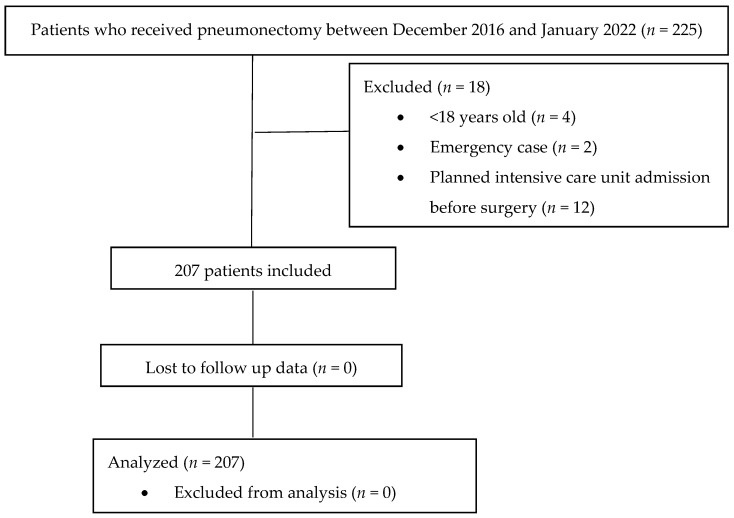
Flow diagram of the study.

A total of 207 patients were included in the study (Figure 1).

### 3.1. Demographic Data

The mean age of the patients was 56.44 ± 11.79 years. Of the patients, 90.3% (*n* = 187) were male, 9.7% (*n* = 20) were female, and 131 patients (63.3%) had a smoking history. Seventy-five patients exhibited comorbidities, including 22 with diabetes mellitus (10.6%), 19 with coronary artery disease (9.2%), 15 with chronic obstructive pulmonary disease (7.2%), 12 with extra-pulmonary malignancy (5.8%), and 7 with hypertension (3.4%). A total of 46 patients (22.2%) received neo-adjuvant treatment. The majority of patients (92.8%) underwent a pneumonectomy via thoracotomy. Standard pneumonectomy was performed in 165 patients (79.7%), intra-pericardial pneumonectomy in 33 patients (15.9%), and tracheal sleeve pneumonectomy in 9 patients (4.4%). A total of 78 patients (37.7%) underwent a right pneumonectomy, while 129 patients (62.3%) underwent a left pneumonectomy. A total of 17 patients (8.2%) required postoperative mechanical ventilation. While 204 patients did not experience intraoperative complications, two patients sustained vascular injury and one exhibited hemodynamic instability. In the postoperative period, the majority of patients (79.7%) did not experience any complications. The most prevalent postoperative complications were cardiac arrhythmia (8.7%), respiratory failure (5.3%), and bronchopleural fistula (3.9%). The median length of hospital stay was 8 days (IQR 6–11 days) and the median length of ICU stay was 1 day (IQR 1–2 days).

### 3.2. Need for ICU Admission After Pneumonectomy

A total of 88 patients (42.5%) were admitted to the ICU. The most common reason for ICU admission was close follow-up (73.9%), other reasons and their rates were as follows: respiratory failure (21.6%), acute renal failure (2.3%), hemodynamic instability (1.1%), and post-resuscitation (1.1%). The demographic and surgical characteristics of ICU-admitted patients are shown in Table 1. It was observed that patients who underwent sleeve or intra-pericardial pneumonectomy were more likely to need ICU admission than those who underwent standard pneumonectomy (*p* = 0.001) (Table 1). The frequency of postoperative complications was higher in ICU-admitted patients than not ICU-admitted patients (9.2% vs. 35.2%, *p* < 0.001) (Table 1). Similarly, cardiac arrhythmia, respiratory failure, and contralateral pneumonia were more common in ICU-admitted patients (*p* = 0.011, *p* < 0.001, *p* = 0.043, respectively) (Table 1). Preoperative serum albumin level was statistically lower, the need for blood transfusion was higher, and the length of hospital stay was longer in ICU-admitted patients than in non-ICU-admitted patients (*p* = 0.017, *p* = 0.020, and *p* = 0.026, respectively) (Table 1). However, other preoperative laboratory values, neo-adjuvant therapy, smoking history, and other factors evaluated including age, gender, comorbidities, duration of surgery, surgical approach, and side of surgery did not exhibit statistical significance as predictors for ICU admission (*p* > 0.05) (Table 1).

### 3.3. Logistic Regression Analysis to Determine Predictors of ICU Admission After Pneumonectomy

As a result of the multiple logistic regression model created to determine the risk factors affecting ICU admission after pneumonectomy using the variables age, gender, serum albumin level, surgical procedure, presence of postoperative complications, and the need for blood transfusion which were found to be significant in single analyses, it was determined that the independent determinants were intra-pericardial pneumonectomy and postoperative complications (OR = 3.46; 95%CI: 1.45–8.23; *p* = 0.005 and OR = 5.10; 95%CI: 2.21–11.79; *p* < 0.001, respectively) (Table 2).

### 3.4. Twenty-Eight-Day Survival After Pneumonectomy

Eight patients (3.9%) died within 28 days following pneumonectomy. The rates of sleeve or pericardial pneumonectomy (*p* = 0.010) and intraoperative vascular injury (*p* = 0.003) were higher in patients who died within 28 days. Additionally, the incidence of postoperative mechanical ventilation (*p* < 0.001), postoperative acute renal failure (*p* = 0.018), sepsis (*p* = 0.008), respiratory failure (*p* < 0.001), and pneumonia (*p* = 0.025) was also higher in these patients. The need for blood transfusion (*p* = 0.047), elevated blood urea nitrogen (*p* = 0.046), and elevated creatinine levels (*p* = 0.004) were found to be higher in patients who died within 28 days compared to those who survived. Nevertheless, other variables did not demonstrate statistical significance and did not suggest an increased risk of 28-day mortality (*p* > 0.05) (Table 3).

### 3.5. Ninety-Day Survival After Pneumonectomy

Fourteen patients (6.8%) died within 90 days following pneumonectomy. It was observed that patients who died within 90 days exhibited higher preoperative NLR values (*p* = 0.019) and serum creatinine levels (*p* = 0.008) and had a greater prevalence of sleeve or intra-pericardial pneumonectomy (*p* = 0.002). In contrast, those who survived exhibited a lower incidence of the need for mechanical ventilation (*p* < 0.001), intraoperative vascular injury (*p* = 0.049), postoperative development of sepsis (*p* < 0.001), respiratory failure (*p* = 0.019), and contralateral pneumonia (*p* = 0.008) than those who did not (Table 4). Other evaluating parameters did not demonstrate statistical significance and did not suggest an increased risk of mortality (*p* > 0.05) (Table 4).

## 4. Discussion

The current study revealed that preoperative WBC count, NAR, LMR, and PLR values did not exhibit associations with ICU admission or survival after pneumonectomy. However, lower albumin levels were linked to a higher likelihood of ICU admission, while higher NLR values were associated with increased 90-day mortality. Additionally, our findings indicated that intra-pericardial pneumonectomy and the presence of postoperative complications were significant predictors of ICU admission.

In thoracic surgery, the ability to predict the need for ICU admission is of utmost importance for thoracic surgeons, anesthesiologists, and intensivists. Respiratory failure requiring mechanical ventilation remains a common cause of emergency ICU admission after lung resection [8]. Previous research reported a reintubation rate of 16% following pneumonectomy [2]. In our study, we observed that 42.5% of patients were admitted to the ICU after pneumonectomy and only 8.2% required mechanical ventilation. In our opinion, the fact that more than half of the patients (73.9%) were admitted to the ICU for follow-up only shows the necessity and importance of preoperative determination of ICU admission for effective use of ICU beds.

All pneumonectomy procedures are associated with postoperative cardiopulmonary complications, leading to an increased need for ICU admission and mortality [2,5,9]. The most common postoperative complications are related to respiratory and cardiac events, including cardiac herniation, hemodynamic fluctuations, and cardiac dysrhythmia. In particular, intra-pericardial and tracheal sleeve pneumonectomy have a higher risk of complications than standard pneumonectomy due to their more invasive nature [2,10]. Blanc et al. showed that specific types of extended pneumonectomy are an independent risk factor for re-intubation after pneumonectomy surgery [2]. Perioperative blood transfusion is another risk factor for clinical outcomes after resection of lung cancer. Kidane et al. demonstrated that perioperative red blood cell use is an independent risk factor for respiratory complications after pneumonectomy and emphasized the importance of restrictive blood practices [11]. Consistent with the literature, we found that cardiac arrhythmias and respiratory failure were the most common postoperative complications. Our study also demonstrated the impact of postoperative complications on ICU admission and mortality. In addition, the need for blood transfusion and tracheal sleeve or intra-pericardial procedures were common in patients admitted to the ICU and were associated with 28-day mortality. Among the laboratory values evaluated, only preoperative serum albumin level was associated with ICU admission after pneumonectomy. However, in logistic regression analysis, only intra-pericardial pneumonectomy and the need for blood transfusion were found to be independent risk factors for ICU admission, whereas albumin was not. Our findings also highlight that patients with intraoperative vascular injury and postoperative renal dysfunction (elevated BUN and creatinine levels) may be at risk for postoperative mortality.

A higher preoperative LMR is linked to a reduced risk of postoperative complications and improved outcomes [12]. Conversely, elevated preoperative NLR and PLR are associated with an increased risk of postoperative complications, a more severe inflammatory response, and poorer prognosis and all of these parameters have been used as prognostic markers in patients undergoing lung cancer surgery [7,12,13,14,15,16,17,18,19]. In recent years, NLR has been identified as a crucial parameter for predicting patient outcomes in solid tumors, including lung cancer, as it reflects the balance between the undesirable effects of neutrophilia and lymphocyte-mediated immunity [13]. In addition, elevated NLR values were used to predict preoperative and postoperative sepsis [20]. Albumin is a negative acute phase reactant, and its serum level decreases under inflammatory conditions. In recent years, some studies have suggested the use of NAR as a prognosis predictor in cancer patients [21,22]. Nevertheless, no previous studies have explored these parameters’ impact on survival after pneumonectomy. In the current study, we observed that preoperative NLR was higher in patients who died within 90 days and preoperative albumin levels were lower in patients who required ICU admission than in those who did not. And these differences were statistically significant. However, no statistically significant difference could be found between preoperative WBC count, NAR, LMR, PLR values and postoperative ICU admission and mortality.

A notable strength of this study, to the best of our knowledge, is its pioneering examination of the inflammation-based variables on the necessity for ICU admission and survival in patients who specifically undergoing pneumonectomy specifically for lung cancer. Although these values have been explored as potential prognostic markers in other contexts, their potential to predict outcomes after pneumonectomy has remained largely uncharted. Nevertheless, it is essential to acknowledge several limitations that should be taken into account. Firstly, this study is retrospective and is conducted at a single center. Secondly, the sample size at 28 and 90 days is relatively modest, so multivariate analysis for 28- and 90-day mortality could not be generated. Thirdly, due to constraints relating to data availability, data quality, data reliability, and the desire to capture the most up-to-date trends and outcomes in pneumonectomy patients, our retrospective analysis is limited to the period spanning from 2016 to 2022. Finally, certain parameters, including preoperative pulmonary function test values, C-reactive protein levels, mechanical ventilation and fluid management strategies, could not be assessed due to a lack of data.

## 5. Conclusions

Intra-pericardial pneumonectomy and the need for blood transfusion during the perioperative period are independent predictors of ICU admission after pneumonectomy. Tracheal sleeve and intra-pericardial procedures, intraoperative and postoperative complications, the need for blood transfusion, preoperative NLR, BUN and creatinine levels may also be potential risk factors for 28- and 90-day mortality. In particular, NLR levels can be easily calculated based on daily complete blood tests, and there is no additional cost. However, to gain a more comprehensive understanding and to better identify patients at increased risk of unfavorable outcomes, further studies should encompass larger patient cohorts and integrate various clinical and laboratory parameters including inflammatory parameters.

## Figures and Tables

**Table 1:** Need for ICU admission after pneumonectomy.

	Need for ICU Admission		
	No (*n* = 119)	Yes (*n* = 88)	*p*-Value
Age (year)	55.59 ± 11.30	57.60 ± 12.39	0.225
Gender (male/female)	107/12	80/8	0.999
Smoking history (no/yes)	37/82	39/49	0.059
Comorbidity			
▪ HT	3	4	0.463
▪ DM	10	12	0.259
▪ COPD	5	10	0.060
▪ CAD	10	9	0.808
▪ Extra-pulmonary malignancy	6	6	0.765
Neo-adjuvant therapy			
▪ No	94	67	0.735
▪ Yes	25	21	
- Chemotherapy	17	14	0.844
- Chemotherapy + Radiotherapy	8	7	0.790
Duration of surgery			
▪ 60–90 min	93	75	0.214
▪ 90–120 min	26	13	
Surgical approach (Thoracotomy/VATS)	107/12	85/3	0.102
Side of surgery (right/left)	42/77	36/52	0.469
Surgical procedure			
▪ Standard pneumonectomy	105	60	
▪ Tracheal sleeve pneumonectomy	2	7	**0.001**
▪ Intra-pericardial pneumonectomy	12	21	
Intraoperative complications			
▪ No	119	85	
▪ Vascular injury	0	2	0.075
▪ Hemodynamic instability	0	1	
Postoperative complications			
▪ No	108	57	**<0.001**
▪ Yes	11	31	
- Bronchopleural fistula	3	5	0.289
- Empyema	5	1	0.244
- Acute renal failure	1	5	0.085
- Sepsis	1	3	0.314
- Cardiac arrhythmia	5	13	**0.011**
- Respiratory failure	0	11	**<0.001**
- Contralateral pneumonia	1	6	**0.043**
Laboratory parameters			
▪ Lymphocyte count (10^3^/µL)	2.05 ± 0.79	2.10 ± 0.86	0.652
▪ Monocyte count (10^3^/µL)	0.83 ± 0.44	0.87 ± 0.38	0.196
▪ Neutrophil count (10^3^/µL)	7.07 ± 3.68	6.97 ± 3.97	0.545
▪ Platelet count (10^3^/µL)	308.16 ± 106.29	319.36 ± 113.98	0.468
▪ Lymphocyte-to-monocyte ratio	2.86 ± 1.50	2.65 ± 1.13	0.598
▪ Platelet-to-lymphocyte ratio	169.44 ± 77.28	181.75 ± 124.90	0.848
▪ Neutrophil-to-lymphocyte ratio	4.07 ± 3.14	4.07 ± 3.75	0.453
▪ Albumin (g/dL)	3.38 ± 0.62	3.15 ± 0.70	**0.017**
▪ Neutrophil-to-albumin ratio	2.22 ± 1.36	2.37 ± 1.42	0.396
▪ White blood cell count (10^3^/µL)	10.12 ± 3.92	10.34 ± 4.68	0.718
▪ Hemoglobin (g/dL)	12.99 ± 1.86	12.70 ± 1.85	0.267
▪ Hematocrit (%)	38.92 ± 5.20	38.46 ± 5.41	0.531
▪ Blood urea nitrogen (mg/dL)	13.67 ± 4.16	14.87 ± 5.59	0.093
▪ Creatinine (mg/dL)	0.83 ± 0.24	0.87 ± 0.28	0.276
Need for blood transfusion	16 (13.6%)	24 (27.3%)	**0.020**
Length of hospital stay (day)	8 (6–9)	9 (6–14)	**0.026**

All data were presented as number of patients (*n*), percentage (%), mean ± standard deviation or median (Q1–Q3). CAD: coronary artery disease; COPD: chronic obstructive pulmonary disease; DM: diabetes mellitus; HT: hypertension; VATS: video-assisted thoracic surgery.

**Table 2:** Logistic regression analysis to determine the risk factors of ICU admission.

	*p*-Value	OR	95%CI for OR
Age	0.381	1.01	0.98–1.05
Gender	0.366	1.70	0.54–5.35
Albumin	0.065	0.63	0.38–1.03
Procedure (standard pneumonectomy)	**0.006**	Ref.	Ref.
Sleeve pneumonectomy	0.066	5.29	0.90–31.20
Intra-pericardial pneumonectomy	**0.005**	3.46	1.45–8.23
Postoperative complications	**<0.001**	5.10	2.21–11.79
The need for blood transfusion	0.072	2.15	0.934–4.94

OR, Odds ratio; CI, confidence interval.

**Table 3:** Twenty-eight-day survival in overall patients after pneumonectomy.

	Alive (*n* = 199)	Died (*n* = 8)	*p*-Value
Age (year)	56.21 ± 11.91	62.38 ± 5.83	0.147
Gender (male/female)	179/20	8/0	0.999
Smoking history (no/yes)	74/125	2/6	0.713
Comorbidity			
▪ HT	6	1	0.245
▪ DM	20	2	0.204
▪ COPD	15	0	0.999
▪ CAD	18	1	0.544
▪ Extra-pulmonary malignancy	12	0	0.999
Neo-adjuvant therapy			
▪ None	156	5	0.380
▪ Yes	43	3	
- Chemotherapy	30	1	0.999
- Chemotherapy + Radiotherapy	13	2	0.107
Duration of surgery			
▪ 60–90 min	161	7	0.999
▪ 90–120 min	38	1	
Surgical approach (Thoracotomy/VATS)	184/15	8/0	0.999
Side of surgery (right/left)	74/125	4/4	0.479
Surgical procedure			
▪ Standard pneumonectomy	162	3	
▪ Tracheal sleeve pneumonectomy	8	1	**0.010**
▪ Intra-pericardial pneumonectomy	29	4	
Intraoperative complications			
▪ No	197	7	
▪ Vascular injury	1	1	**0.003**
▪ Hemodynamic instability	1	0	
Need for mechanical ventilation	9 (4.5%)	8 (100%)	**<0.001**
Postoperative complications			
▪ No	164 (82.4%)	1 (12.5%)	**<0.001**
▪ Yes	35 (17.6%)	7 (87.5%)	
- Bronchopleural fistula	7	1	0.274
- Empyema	6	0	0.999
- Acute renal failure	4	2	**0.018**
- Sepsis	2	2	**0.008**
- Cardiac arrhythmia	16	2	0.146
- Respiratory failure	7	4	**<0.001**
- Contralateral pneumonia	5	2	**0.025**
Laboratory parameters			
▪ Lymphocyte count (10^3^/µL)	2.09 ± 0.82	1.76 ± 0.67	0.262
▪ Monocyte count (10^3^/µL)	0.85 ± 0.42	0.90 ± 0.34	0.683
▪ Neutrophil count (10^3^/µL)	6.96 ± 3.66	8.60 ± 6.54	0.237
▪ Platelet count (10^3^/µL)	314.84 ± 110.70	265.13 ± 59.10	0.209
▪ Lymphocyte-to-monocyte ratio	2.79 ± 1.36	2.17 ± 1.32	0.205
▪ Platelet-to-lymphocyte ratio	174.75 ± 100.98	172.84 ± 85.03	0.958
▪ Neutrophil-to-lymphocyte ratio	4.02 ± 3.35	5.53 ± 4.46	0.219
▪ Albumin (g/dL)	3.29 ± 0.65	3.22 ± 1.02	0.796
▪ Neutrophil-to-albumin ratio	2.26 ± 1.36	2.87 ± 1.99	0.512
▪ White blood cell count (10^3^/µL)	10.17 ± 4.15	11.35 ± 6.56	0.442
▪ Hemoglobin (g/dL)	12.90 ± 1.87	12.03 ± 1.39	0.198
▪ Hematocrit (%)	38.78 ± 5.31	37.21 ± 4.59	0.410
▪ Blood urea nitrogen (mg/dL)	14.05 ± 4.82	17.53 ± 4.62	**0.046**
▪ Creatinine (mg/dL)	0.84 ± 0.25	1.11 ± 0.38	**0.004**
Need for blood transfusion	36 (18.1%)	4 (50%)	**0.047**
Length of hospital stay (day)	8 (6–11)	9 (4–23)	0.098

All data were presented as number of patients (*n*), mean ± standard deviation, percentage (%) or median (Q1–Q3). All laboratory values were assessed one day before surgery. CAD: coronary artery disease; COPD: chronic obstructive pulmonary disease; DM: diabetes mellitus; HT: hypertension; VATS: video-assisted thoracic surgery.

**Table 4:** Ninety-day survival in overall patients after pneumonectomy.

	Alive (*n* =193)	Died (*n* =14)	*p*-Value
Age (year)	56.35 ± 11.54	57.71 ± 15.19	0.677
Gender (male/female)	174/19	13/1	0.999
Smoking history (no/yes)	72/121	4/10	0.580
Comorbidity			
▪ HT	6	1	0.394
▪ DM	19	3	0.175
▪ COPD	15	0	0.605
▪ CAD	18	1	0.999
▪ Extra-pulmonary malignancy	12	0	0.999
Neo-adjuvant therapy			
▪ None	150	11	0.999
▪ Yes	43	3	
- Chemotherapy	30	1	0.699
- Chemotherapy + Radiotherapy	13	2	0.269
Duration of surgery			
▪ 60–90 min	155	13	0.476
▪ 90–120 min	38	1	
Surgical approach (Thoracotomy/VATS)	178/15	14/0	0.605
Side of surgery (right/left)	70/123	8/6	0.154
Surgical procedure			
▪ Standard pneumonectomy	159	6	
▪ Tracheal sleeve pneumonectomy	7	2	**0.002**
▪ Intra-pericardial pneumonectomy	27	6	
Intraoperative complications			
▪ No	191	13	
▪ Vascular injury	1	1	**0.049**
▪ Hemodynamic instability	1	0	
Need for mechanical ventilation (no/yes)	187/6	3/11	**<0.001**
Postoperative complications			
▪ No	162 (83.9%)	3 (21.4%)	**<0.001**
▪ Yes	31 (16.1%)	11 (78.6%)	
- Bronchopleural fistula	7	1	0.435
- Empyema	6	0	0.999
- Acute renal failure	4	2	0.055
- Sepsis	0	4	**<0.001**
- Cardiac arrhythmia	15	3	0.109
- Respiratory failure	4	7	**<0.001**
- Contralateral pneumonia	2	5	**<0.001**
Laboratory parameters			
▪ Lymphocyte count (10^3^/µL)	2.10 ± 0.82	1.72 ± 0.73	0.093
▪ Monocyte count (10^3^/µL)	0.85 ± 0.42	0.78 ± 0.33	0.525
▪ Neutrophil count (10^3^/µL)	6.93 ± 3.67	8.38 ± 5.29	0.168
▪ Platelet count (10^3^/µL)	313.41 ± 111.05	306 ± 88.22	0.811
▪ Lymphocyte-to-monocyte ratio	2.80 ± 1.37	2.41 ± 1.17	0.307
▪ Platelet-to-lymphocyte ratio	171.47 ± 96.34	218 ± 140.90	0.880
▪ Neutrophil-to-lymphocyte ratio	3.93 ± 3.17	6.13 ± 5.47	**0.019**
▪ Albumin (g/dL)	3.30 ± 0.6	2.97 ± 1.0	0.067
▪ Neutrophil-to-albumin ratio	2.23 ± 1.35	3.03 ± 1.73	0.061
▪ White blood cell count (10^3^/µL)	10.15 ± 4.18	11.01 ± 5.21	0.468
▪ Hemoglobin (g/dL)	12.89 ± 1.87	12.50 ± 1.72	0.453
▪ Hematocrit (%)	38.76 ± 5.30	38.22 ± 5.26	0.713
▪ Blood urea nitrogen (mg/dL)	14.07 ± 4.82	15.72 ± 5.13	0.219
▪ Creatinine (mg/dL)	0.84 ± 0.25	1.03 ± 0.30	**0.008**
Need for blood transfusion	158/35	9/5	0.152
Length of hospital stay (day)	9.57 ± 5.48	20.36 ± 21.16	**<0.001**
	8 (6–10)	11 (6–29)	

All data were presented as number of patients (*n*), percentage (%), mean ± standard deviation or median (Q1–Q3). All laboratory values were assessed one day before surgery. CAD: coronary artery disease; COPD: chronic obstructive pulmonary disease; DM: diabetes mellitus; HT: hypertension; VATS: video-assisted thoracic surgery.

## Data Availability

Data are available upon request from the authors.
